# Which nasopharyngeal cancer patients need adaptive radiotherapy?

**DOI:** 10.1186/s12885-018-5159-y

**Published:** 2018-12-10

**Authors:** Yu-Chang Hu, Kuo-Wang Tsai, Ching-Chih Lee, Nan-Jing Peng, Ju-Chun Chien, Hsin-Hui Tseng, Po-Chun Chen, Jin-Ching Lin, Wen-Shan Liu

**Affiliations:** 1 Department of Radiation Oncology, Kaohsiung Veterans General Hospital, Kaohsiung, Taiwan; 2 School of Medicine, National Defense Medical Center, Taipei, Taiwan; 3 Department of Medical Education and Research, Kaohsiung Veterans General Hospital, Kaohsiung, Taiwan; 4 Department of Chemical Biology, National Pingtung University of Education, Pingtung, Taiwan; 5 Department of ENT, Kaohsiung Veterans General Hospital, Kaohsiung, Taiwan; 6 Department of Nuclear Medicine, Kaohsiung Veterans General Hospital, Kaohsiung, Taiwan; 7 Department of Radiation oncology, Pingtung Christian Hospital, Pingtung, Taiwan; 8 Graduate Institute of Bioresources, National Pingtung University of Science and Technology, Pingtung, Taiwan; 9 Department of Radiation Oncology, Taichung Veterans General Hospital, Taichung, Taiwan

**Keywords:** Nasopharyngeal cancer, Radiotherapy, Adaptive radiotherapy, Intensity-modulated radiotherapy, Volumetric modulated arc radiotherapy

## Abstract

**Background:**

Adaptive radiotherapy (ART) has potential benefits in patients with nasopharyngeal cancer (NPC). This retrospective study aimed to identify the factors favoring ART.

**Materials and methods:**

Forty NPC patients were retrospectively included in this study. All patients received two-phase, volumetric modulated arc radiotherapy (VMAT) and underwent a second computed tomography (CT) for the phase II ART. We generated phantom, non-ART plans by a hybrid method for comparison with ART plans. A paired *t-*test was used to evaluate the dose differences between these two plans. A subgroup analysis through a paired *t*-test was used to evaluate the factors favoring ART.

**Results:**

The second CT images were captured at the median 22 fractions. The median total dose of the planning target volume-one (PTV-1) was 72 Gy, and the phase II dose was 16 Gy. The volumes of the ipsilateral parotid gland (23.2 vs. 19.2 ml, *p* <  0.000), contralateral parotid gland (23.0 vs. 18.4 ml, *p* <  0.000), clinical target volume-1 (CTV-1, 32.2 vs. 20.9 ml, *p* <  0.000), and PTV-1 (125.8 vs. 107.3 ml, *p* <  0.000) all shrunk significantly between these two CT simulation procedures. Among the nearby critical organs, only the ipsilateral parotid gland displayed significant dose reduction by the ART plan (5.3 vs. 6.0 Gy, *p* = 0.004). Compared to the phantom plan, the ART could significantly improve the PTV-1 target volume coverage of D_98_ (15.4 vs. 12.3 Gy, *p* < 0.000). Based on the D_98_ of PTV-1, the factors of a large initial weight (> 60 kg, *p* < 0.000), large body mass index (BMI) (> 21.5, *p* < 0.000), obvious weight loss (> 2.8 kg, *p* < 0.000), concurrent chemoradiotherapy (*p* < 0.000), and stages III–IV (*p* < 0.000) favored the use of ART.

**Conclusions:**

ART could significantly reduce the mean dose to the ipsilateral parotid gland. ART has dosimetrical benefit for patients with a heavy initial weight, large BMI, obvious weight loss, concurrent chemoradiotherapy, and cancer in stages III–IV.

## Background

Nasopharyngeal cancer is an endemic disease in southern China, with an annual incidence of 30 cases per 100,000 persons [1]. In Taiwan, its annual incidence is approximately 13 cases per 100,000 persons [2]. In the past three decades, treatment outcomes for NPC have significantly improved through more accurate staging, improved radiotherapy techniques, and the administration of chemotherapy [1, 3]. Intensity-modulated radiotherapy (IMRT) is widely used because of its efficiency and improvement in volume coverage [1, 3]. IMRT treatments deliver high doses that conform better to targets and further lower the doses to the surrounding critical organs compared with two- or three-dimensional radiotherapy [3, 4]. Prospective randomized studies proved that IMRT contributed higher quality of life and better salivary preservation than three-dimensional radiotherapy [5–7]. However, because of the high complexity of the anatomical structures surrounding nasopharyngeal primary tumors and metastatic neck lymph nodes, IMRT introduces a high dose gradient between tumors and the nearby critical organs [8]. In this high gradient region, any inaccuracy of positional set-up or anatomical changes may induce of higher doses to nearby organs or lower doses to targets [9, 10]. In contrast with non-ART radiotherapy (including 3D conformal radiotherapy, IMRT and VMAT), ART adapt the dose distribution to target and critical organs according to new CT scan images acquired during the treatment [9]. Hence, it has potential benefit when there were significant changes of body contour or tumor volume during radiotherapy.

Previous studies confirmed that the volumes of tumors and normal organs shrunk significantly during radiotherapy in NPC and other head-and-neck cancers [9, 11–14]. In NPC patients, many critical organs (like parotid glands, optic chiasm, brainstem and spinal cord) locate just adjacent or very close to the tumor. When the tumor volume reduction happened, these critical organs may move into the original high dose region. So, the volume reduction of tumor may significantly affect the doses to the surrounding organs. Under such circumstances, a lack of replanning leads to underestimate of doses to normal organs [9, 11, 13]. As for the target volume, the ART may prevent under dose compared to the situation of without ART [11, 15]. These circumstances are caused by changes in the body contour, tumor shrinkage, and the shifting of tumor position in patients in head and neck cancer patients [11, 13, 15, 16]. For example, Zhao et al. [11] reported that replanning yielded a more favorable D_99_ to the clinical target volume (CTV; *p* = 0.034) than the initial plan. Bhide et al. [15] found the doses for the CTV and planning target volume (PTV) were reduced significantly (*p* < 0.05) if they were evaluated 2 weeks after radiotherapy. Not only was the dose coverage affected, but Luo [17] and Chen [18] also reported that patients with head-and-neck cancer were advantaged by ART with an increasing rate of local control. However, ART requires substantial manpower resources. It would be highly beneficial if we could determine which patients require this procedure before or during radiotherapy. Only a few studies have focused on this important concern [19, 20]. Hence, this study aimed to identify the factors favoring the use of ART for NPC patients as judged by a dosimetric method.

## Methods

### Patients

This study included nasopharyngeal cancer patients received ART radiotherapy from March 2013 to December 2015. The staging system employed was that described in the *American Joint Committee on Cancer’s Cancer Staging Manual, 7th Edition* [21]. The diagnosis of NPC was confirmed by nasopharyngeal biopsy, and the clinical stage was evaluated by clinical staging examinations, including magnetic resonance imaging (MRI), chest X-ray, liver sonography, and bone scan. The institutional review board approved this study.

### Adaptive radiotherapy

The radiotherapy protocol was a two-phase treatments and the phase II adaptive plan was calculated based on the second CT images scanned during radiotherapy. All patients were immobilized using a thermoplastic mask and a localizer frame system (U-Plast thermoplastic mask, Orfit Inc). All patients received contrast injections during CT scanning with a slice thickness of 3 mm. The second CT was scheduled at least one week before the starting of phase II treatment. Two practical reasons caused this arrangement. First, physician need to schedule their time for re-delineation of target and normal organs, and, physicists need time for processing the plan calculation. The dose prescription of phase I were 56 Gy/28 fractions for high risk volume (PTV-1) plus intermediate risk volume (PTV-2) and 50.4 Gy/28 fractions for low risk volume (PTV-3), simultaneously. The phase II adaptive plan was administered at least 14 Gy/7 fractions to PTV-1 only. Hence, the total dose of PTV-1 was 70 Gy/35 fractions at least. The definition of gross tumor volume (GTV) was the gross nasopharyngeal tumor, enlarged retropharyngeal lymph nodes (≥ 0.5 cm), and enlarged neck lymph nodes (≥ 1.5 cm). This GTV was delineated according to the fusion images of a gadolinium-contrast, T1 axial MRI and contrast-enhanced CT images. The CTV-1 was extended 3–4 mm from GTV according to the distance of nearby critical organs. The definition of CTV-2 was the primary tumor site and intermediate risk lymphatic regions. This target volume included the skull base; inner half of the clivus; inferior third of the sphenoid sinus; retropharyngeal regions; pterygoid fossa; inner margin of the lateral pterygoid muscle; posterior fourth of the nasal cavity; 5 mm above the posterior maxillary sinus wall; and bilateral levels II, III, and Va of the neck lymphatic regions. The CTV-3 was defined as the bilateral level IV, level Vb, and bilateral supraclavicular fossa for low-risk regions. Through a planning system (Eclipse Planning System, version 13, Palo Alto, CA, USA), the PTV-1, PTV-2, and PTV-3 were automatically extended from the CTV-1, CTV-2, and CTV-3, respectively. These extensions were 4–5 mm according to the distance from critical organs, such as the brainstem and optic chiasm. The CTV-1 of the adaptive plan was delineated by the second CT images. Radiotherapy was delivered by the Volumetric Arc Therapy (VMAT) method. Two to three partial arcs were designed according to the difficulty of protecting the nearby critical organs. The treatment plans were calculated by the above planning system and delivered by a Varian Clinac iX linear accelerator. There was no break between phases I and II of the treatments.

### Phantom non-ART plans

To evaluate the role of ART and search for the factors indicating ART, a phantom plan was created to simulate the non-ART situation. This phase II phantom non-ART plan was performed by a hybrid plan method [13]. This comprised the following two steps. First, the simulated phase II plan was calculated according to the first CT images with the initial PTV-1 target volumes. Second, this beam configuration was applied to the anatomy and target volumes from the second CT images for generating the phantom plan.

### Chemotherapy

Nine patients received neoadjuvant chemotherapy before concurrent chemoradiotherapy (CCRT); this was comprised of 80 mg/m^2^ of cisplatin for 1 day and 1000 mg/m^2^ of 5-fluorouracil for 5 days every 3 weeks for 2 to 3 cycles. All patients received CCRT; 33 patients received weekly cisplatin of 40 mg/m^2^ and 7 patients received cisplatin of 80 mg/m^2^ every 3 weeks.

### Body weight, volume, and dosimetry comparisons

We compared the body weight, volume of the parotid gland, and the PTV-1 target volumes between the first and second CT scans. According to *ICRU Report 83* [22], we compared various doses to the target volumes and normal organs between the ART and phantom plan. These target volume doses were D_98_, D_95,_ and D_50_ of PTV-1. Following the suggestion of *ICRU Report 83*, the D_2_, D_max_, and D_mean_ of the various normal organs were compared.

### Factors favoring adaptive radiotherapy

Factors were evaluated by comparision between ART and non-ART phantom plans. These factors included initial body weight (60 kg), BMI (21.5), body weight loss (2.8 kg), chemotherapy (neoadjuvant and CCRT vs. CCRT), and stage (stage II vs. III–IV). The cutoff levels of the continuous variables were assigned as the upper or lower 25% percentile.

### Statistical analysis

We compared the changes in body weight, target volumes, and parotid gland volumes between the first and second CT simulations by a paired *t-*test. Likewise, we used a paired *t-*test to compare the various dosimetry factors between the ART and non-ART plans. Factors favoring ART were analyzed by pair t-test between ART and non-ART phantom plans. The statistical software was SPSS version 20.

## Results

### Patient characteristics

Forty-five patients received ART treatment. To reduce possible confounding factors, we excluded four patients who received concurrent biochemotherapy and one patient who received daily treatment exceeding 2.2 Gy. The characteristics of the patients are listed in Table 1. The stage distributions were stage II in 6, stage III in 23, and stage IV in 11 patients. The second CT simulations were performed at the median 22 fractions (Table 1). The phase II adaptive plan was administered with a median dose of 16.0 Gy, ranging from 14.0 to 18.0 Gy. The median total dose of PTV-1 was 72.0 Gy and ranged from 70 to 74 Gy. There were three T4 patients who received 18 Gy in 9 fractions for phase II plans (boost plans). The other 37 patients received 14–16 Gy/7–8 fractions for phase II treatments.

**Table 1:** Patient characteristics (*n* = 40)

Variables	Number	Median (mean ± SD)
Female/male	11 / 29	
Age (y/o)		48.5 (49.1 ± 11.8)
T Stage*		
T1/T2	8/5	
T3/4	19 / 8	
N Stage*		
N0/1	3 / 9	
N2/N3a/N3b	23 / 1 / 4	
Stage*		
I/II/III	0 / 6 / 23	
IVa/IVb	6 / 5	
CCRT+neo-adj / CCRT	31 / 9	
Fractions of 2nd CT scan		22.0 (22.2 ± 1.8)
ART boost plans (Gy)		16.0 (15.6 ± 1.6)
Total PTV-1 dose (Gy)		72.0 (71.6 ± 1.2)

Abbreviations: *CCRT* concurrent chemoradiotherapy, *neo-adj* neoadjuvant chemotherapy, *ART* adaptive radiotherapy, *PTV-1,*planning target volume of CTV-1. *According to the *AJCC Cancer Staging Manual, 7th Edition*

### Body weight, volume, and dosimetry comparisons

The mean body weight decreased significantly from the first to the second CT simulation (68.4 vs. 64.1 kg, *p* < 0.000). The volumes of CTV-1 shrunk significantly from 32.2 to 17.9 mL (*p* < 0.000), and the corresponding PTV-1 also shrunk significantly from 125.8 to 107.3 mL (*p* < 0.000). The volumes of the ipsilateral and contralateral parotid glands were all significantly reduced from 23.2 to 19.2 ml (*p* < 0.000) and 23.0 to 18.4 ml (*p* < 0.000), respectively (Table 2).

**Table 2:** Body weight and volume changes evaluated by CT simulations (*n* = 40)

Variables	1st CT (mean ± SD)	2nd CT (mean ± SD)	Change (%)	*p*-value
Body weight (kg)	68.4 ± 13.3	64.1 ± 12.6	− 6.3	< 0.000
CTV-1 (ml)	32.2 ± 22.7	20.9 ± 18.5	−35.1	< 0.000
PTV-1 (ml)	125.8 ± 53.2	107.3 ± 43.7	− 14.7	< 0.000
Parotid-1 (ml)	23.2 ± 8.0	19.2 ± 8.0	− 17.2	< 0.000
Parotid-2 (ml)	23.0 ± 7.8	18.4 ± 8.1	− 20.0	< 0.000

Abbreviations: *CT* computed tomography, *CTV-1* clinical target volume-1, *PTV-1* planning target volume of CTV-1

The D_98_ (*p* < 0.000) and D_95_ (*p* = 0.001) of the PTV-1 in the ART plans were significantly higher than in the phantom plans (15.4 Gy vs. 12.3 Gy and 15.6 Gy vs. 13.8 Gy, respectively; Table 3). There was no significant difference in D_50_ of the PTV-1 between these two plans. As for the doses of the nearby normal organs, ART could only significantly benefit the ipsilateral parotid glands (5.3 Gy vs. 6.0 Gy, *p* = 0.004). Other normal organs did not display significant differences between the ART and phantom plans (Table 3).

**Table 3:** Dosimetry differences between ART and phantom non-ART plans in various target volumes and organs at risk (*n* = 40)

Variables	ART* (Gy; mean ± SD)	Phantom plan (Gy; mean ± SD)	Change (%)	*p*-value
PTV-1, D_98_	15.4 ± 1.6	12.3 ± 3.7	20.1	< 0.000
PTV-1, D_95_	15.6 ± 1.6	13.8 ± 3.2	13.0	0.001
PTV-1, D_50_	16.0 ± 1.7	15.9 ± 1.6	0.6	0.071
Brainstem, D_2_	6.6 ± 1.8	6.6 ± 1.7	−0.0	0.990
Cord, D_max_	6.0 ± 1.3	6.4 ± 1.4	−6.3	0.243
Chiasm, D_max_	1.8 ± 2.0	2.1 ± 2.8	−16.5	0.241
Chiasm, D_mean_	1.3 ± 1.3	1.5 ± 2.2	−13.3	0.285
Optic N, D_max_	2.3 ± 2.9	2.6 ± 3.5	−13.0	0.390
Optic N, D_mean_	1.0 ± 0.8	1.2 ± 1.2	−20.0	0.282
Cochlear, D_max_	8.3 ± 2.8	8.6 ± 2.7	−3.6	0.501
Cochlear, D_mean_	6.1 ± 2.0	6.0 ± 2.0	1.7	0.899
Parotid-1, D_mean_	5.3 ± 1.6	6.0 ± 2.3	−11.7	0.004
Parotid-2, D_mean_	4.2 ± 1.3	4.3 ± 1.4	−2.3	0.617

*Mean ART PTV-1 prescribed dose = 15.6 ± 1.6 Gy

Abbreviations: *ART* adaptive radiotherapy, *D*_*x*_ dose to *x*% of the target volume *D*_*max*_ maximal dose, *D*_*mean*_ mean dose, *parotid-1* ipsilateral parotid glands, *parotid-2* contralateral parotid glands

### Factors in favor of ART

Based on the dosimetry criteria of the D_98_ of PTV-1, the factors of a body weight exceeding 60 kg (15.5 vs. 12.1 Gy, *p* < 0.000), BMI greater than 21.5 (15.1 vs. 13.7 Gy, *p* < 0.000), body-weight loss greater than 2.8 kg (15.4 vs. 12.5 Gy, *p* < 0.000), CCRT (15.5 vs. 12.2 Gy, *p* < 0.000), and stage III–IV (15.7 vs. 12.5 Gy, *p* < 0.000) significantly favored the ART plans instead of the phantom non-ART plans (Table 4).

**Table 4:** Dosimetric comparisons between adaptive and phantom plans (*n* = 40)

Factors/plans (n)	D_98_ of PTV-1 (Gy; mean ± SD)	*p*-value	D_mean_ of Parotid-1 (Gy; mean ± SD)	*p*-value
BW < 60 kg		0.053		0.049
ART(11)	15.0 ± 1.0		6.3 ± 2.4	
Phantom(11)	13.0 ± 3.6		7.3 ± 2.9	
BW > 60 kg		0.000		0.040
ART(29)	15.5 ± 1.8		4.8 ± 0.9	
Phantom(29)	12.1 ± 3.8		5.3 ± 1.3	
BMI < 21.5		0.119		0.060
ART(11)	15.1 ± 0.9		5.9 ± 2.4	
Phantom(11)	13.7 ± 3.2		7.5 ± 3.1	
BMI > 21.5		0.000		0.034
ART(29)	15.4 ± 1.8		4.9 ± 1.1	
Phantom(29)	11.8 ± 3.8		5.4 ± 1.7	
BW loss < 2.8 kg		0.051		0.079
ART(10)	15.2 ± 1.4		5.1 ± 1.2	
Phantom(10)	12.6 ± 3.6		5.4 ± 1.6	
BW loss > 2.8 kg		0.000		0.020
ART(30)	15.4 ± 1.7		5.5 ± 2.3	
Phantom(30)	12.5 ± 3.2		7.1 ± 3.4	
Neo-adjuvant C/T		0.105		0.081
ART(9)	14.9 ± 1.9		5.9 ± 2.6	
Phantom(9)	12.5 ± 4.2		6.5 ± 3.3	
CCRT		0.000		0.015
ART(31)	15.5 ± 1.5		4.9 ± 1.1	
Phantom(31)	12.2 ± 3.6		5.7 ± 1.9	
Stage II†		0.069		0.671
ART(6)	13.3 ± 1.7		4.3 ± 0.9	
Phantom(6)	11.3 ± 2.6		4.1 ± 1.1	
Stage III/IV†		0.000		0.002
ART(34)	15.7 ± 1.3		5.3 ± 1.6	
Phantom(34)	12.5 ± 3.9		6.2 ± 2.3	

†According to the *AJCC Cancer Staging Manual, 7th Edition*

Abbreviations: *PTV-1* planning target volume of CTV-1, *D*_*98*_ dose to 98% of the target volume, *D*_*mean*_ mean dose, *BW* body weight, *ART* adaptive radiotherapy, *BMI* body mass index, *C/T* chemotherapy, *CCRT* concurrent chemoradiotherapy, *parotid-1* ipsilateral parotid gland

As for the effect on reducing the ipsilateral parotid dose, ART had a significantly lower dose than the phantom non-ART plan in the CCRT (4.5 vs. 4.9 Gy, *p* = 0.043) and stage III–IV groups (4.8 vs. 5.3 Gy, *p* = 0.007; Table 4).

## Discussion

Up to now, it was difficult to conclude what is the optimal time/fraction to perform adaptive replanning by a second CT simulation during radiotherapy for head-and-neck cancer patients. For example, Wang et al. [16] suggested replanning before the 25th fraction. Bhide et al. [15] performed an observation study for evaluation of volume changes by weekly CT scan for 5 weeks. They found that the volumes changed continuously during this period, but the most significant differences were in week two. Jin [9] suggested that the protection of the parotid gland would benefit from replanning after 30 Gy. Based on these studies, the schedules of second CT scan were suggested from 10 to 25 fractions of treatment. Some researchers performed adaptive replanning by clinical demand, such as loss of mask fixation, weight loss (> 15%), or shrinkage of the tumor [11, 13, 20]. In this study, the replanning CT scan was scheduled at least one week before the starting of phase II treatment (median fractions of 22.0, mean 22.2 ± 1.8). Under these circumstances, the CTV-1 shrunk significantly, from 32.2 to 20.9 mL (*p* < 0.000), and the PTV-1 also decreased significantly, from 125.8 to 107.3 ml (*p* < 0.000). The mean body weight significantly decreased from 68.4 kg to 64.1 kg (*p* < 0.000). The volume of the ipsilateral and contralateral parotid glands shrunk significantly, from 23.2 to 19.2 ml (*p* < 0.000) and 23.0 to 18.4 ml (*p* < 0.000), respectively (Table 2). Many studies proved that the weight, volume of tumor, and volume of the parotid glands would change significantly during radiotherapy or CCRT [11–14, 19, 23–26]. Nevertheless, these weight and volume changes led to the issue of whether these contour deviations induced significant dose deviations in the organs at risk or target.

In this study, most of the normal organs had lower doses in the ART plans compared to phantom plans (Table 3). However, only the ipsilateral parotid gland achieved a significant dose reduction in ART plans (5.3 Gy vs. 6.0 Gy, *p* = 0.004). Most of the prior studies had similar results; ART contributed to significantly lowering the doses to the parotid glands (bilateral or ipsilateral) compared to the phantom plans (i.e., simulated non-adaptive) [9, 13–16, 24]. For example, Brown [19] found that the mean dose to the ipsilateral parotid gland had significant differences between the original and delivered plans (42.3 vs. 43.9 Gy, *p* < 0.05). Jin [9] found significant differences between the original and delivered doses for the bilateral parotid glands. Zhang [24] demonstrated a significant reduction of the bilateral parotid gland’s mean dose when replanning at the fifth week. There are two reasons for the benefit to parotid gland protection by replanning. First, many studies found the volume of the parotid glands shrunk significantly during radiotherapy [9, 11, 13, 15, 25]. Second, weight loss that causes contour changes is common in head and neck radiotherapy [13, 19, 26, 27]. Yang et al. found that patients who received IMRT with replanning (ART) had significantly better quality of life compared to those without replanning [28]. However, further study is needed to understand the clinical benefit from dose reduction to the parotid glands by ART. As for other organs at risk, it remains a controversial issue whether ART could significantly reduce the doses to the spinal cord and brain stem. Hansen [13], Bhide [15], and Wang [16] found that ART could significantly reduce the maximum doses to the spinal cord and brain stem. However, Jin [9], Zhao [11], and Wu [14] did not prove a significant dose reduction by replanning. We found that ART did not reduce the D_2_ of brainstem or the D_max_ of the spinal cord (Table 3). Wu proposed the position of these organs do not change during radiotherapy and, hence, may not benefit from ART [14].

At the median dose of 16.0 Gy in the phase II plans, the D_98_ and D_95_ of PTV-1 in the ART plans were significantly higher than in the phantom plans (15.4 vs. 12.3 Gy, *p* < 0.001; 15.6 vs. 13.8 Gy, *p* = 0.001; Table 3). Our findings suggest that the target dose would benefit from ART. These results were similar to those of Hansen et al. [13], Bhide et al. [15], and Wang et al. [16]. Hansen et al. found the D_95_ of PTV-GTV decreased significantly (*p* = 0.02) in 92% of patients if not replanned [13]. Wang et al. [16] reported that the dose of CTV-1 increased significantly by 4.91% (*p* = 0.024) in replanning for nasopharyngeal patients. However, some studies did not confirm the dosimetry benefit of ART for target volumes [9, 14, 24]. These opposing results may be attributed to different dosimetry endpoints or study design, such as different times of replanning. For example, most of the negative reports used D_95_/D_90_ of GTV or CTV instead of PTV [9, 24]. According to the suggestions from *ICRU Report 62* and *ICRU Report 83* [22, 29], we considered it appropriate to examine the dosimetry endpoint by PTV. Luo et al. found that nasopharyngeal cancer patient received ART could yielded significantly better loco-regional progression free survival than non-ART group (97.2% vs. 88.1%, *p* = 0.022) [17]. Chen et al. demonstrated that ART could benefit the loco-regional control survival in head and neck cancer patients (88% vs. 79%, *p* = 0.01) but not overall survival (73% vs. 79%, *p* = 0.55) [18].

ART requires substantial manpower, and it would be highly beneficial if we could identify the patients who require this procedure in advance. Based on our results of the dosimetry differences between ART and phantom plans (Table 3), we used the D_98_ of PTV-1 as the suggested criteria for indicating ART and the mean dose to the ipsilateral parotid gland as the parotid-protection criteria for indicating ART. We found that the D_98_ of PTV-1 could significantly improve for those patients who exceeded the lower 25% percentile of initial body weight (> 60 kg), BMI (> 21.5), and weight loss (> 2.8 kg; Table 4). Brown et al. published one of the few studies to determine the predictors of ART [19]. They concluded that a larger initial weight (> 100 kg) was a high-risk factor indicating ART for patients with NPC. However, they included only 12 nasopharyngeal cancer patients. There are two reasons that explain the differences between this and Brown’s results. First, the body statures in these two studies should be quite different. In this study, the mean initial weight was 68.4 ± 13.3 kg with a maximal weight of 90.8 kg. Second, the statistical method was different. Brown et al. used a logistic regression model, and we used a paired *t*-test. Interpreting these two studies, we suggest considering ART for NPC patients who have heavy weights or high BMIs. However, it may not appropriate to use the cutoff level of 60 kg or 100 kg directly because the distribution of weight may be different in different hospitals or countries. As for the factor of weight loss, Tan et al. [26] found that weight loss correlated with target volume reductions. Gregoire et al. [30] suggested that ART can be considered for anatomical changes. Chen et al. [27] found that weight loss led to a significant increase in the PTV of primary tumor volume doses (1.9–2.9%). Altogether, obvious weight loss (exceeding 2.8 kg in this study) should suggest the use of ART.

CCRT with or without an adjuvant chemotherapy is now the standard of care for nasopharyngeal cancer patients [31]. Many studies tried to evaluate the role of neoadjuvant chemotherapy before CCRT [32]. In this study, there were nine patients who received neoadjuvant chemotherapy prior to CCRT due to clinical considerations, like a locally advanced status. It is unknown whether ART has an equal role in CCRT with or without neoadjuvant chemotherapy patients. We found ART replanning had significantly better target coverage (D_98_ of PTV-1) compared to the phantom plans only in the CCRT groups (15.5 vs. 12.2 Gy, *p* < 0.000) but not in the neoadjuvant group (14.9 vs. 12.5 Gy, *p* = 0.105; Table 4). Tan et al. demonstrated the tumor volume reduction rates were higher in the CCRT groups (42.6%) than the neoadjuvant groups (35.1%). The three-dimensional displacements were larger in the concurrent groups than the neoadjuvant groups [26]. In historical data, the response rate of neoadjuvant chemotherapy in NPC patients was approximately 80% [32]. Hence, the reduction of the tumor volume during radiotherapy would be more significant in CCRT groups than the neoadjuvant groups. A lower reduction in tumor volume results in lower dose variation during radiotherapy [27]. As a result, patients who receive neoadjuvant chemotherapy have less dose deviation during radiotherapy, and the benefit of ART would be less. To the best of our knowledge, this is the first study to disclose the role of ART in comparison with CCRT and neoadjuvant patients. As distinct from stage II (13.3 vs. 11.3 Gy, *p* = 0.069), ART plans provide significantly better target coverage (D_98_ of PTV-1) than phantom plans in patients with stage III and IV patients (15.7 vs. 12.5 Gy, *p* < 0.000). We excluded IVc disease; our stage III and IV diseases were comprised of T3/N2 to T4/N3 status. These results are similar to some previous studies. For example, Brown [19] reported the N2/N3 status is a high risk for ART; Zhao [11] suggested replanning (ART) for T3–4 and N2–3 patients.

Some limitations should be noted in this study. The patient’s weight and height are quite different in each country and hospital. Therefore, it is suggested that these data be applied with cautious. The planning method is another possible limitation. Many scenarios could change the results of VMAT/IMRT plans, such as the weighting factors of different normal organs and the priority of dose coverage of the target volumes. We maintained the same principles between ART and phantom plans to minimize the situation. Finally, this study was drawn by dosimetric advantage but not clinical results.

## Conclusion

For NPC patients who received two phases of VMAT (IMRT) treatment, this retrospective study revealed that adaptive planning could significantly improve the dose coverage (D_98_ and D_95_) of tumors and reduce the dose to the ipsilateral parotid gland in phase II plan. The factors of a larger initial weight, BMI, weight loss, CCRT, and cancer in stages III–IV could be considered for using ART procedure which could provide dosimetric advantage.

## Abbreviations

ART: Adaptive radiotherapy
CCRT: Concurrent chemoradiotherapy
CT: Computed tomography
CTV: Clinical target volume
GTV: Gross tumor volume
IMRT: Intensity-modulated radiotherapy
PTV: Planning target volume
VMAT: Volumetric modulated arc radiotherapy
